# A 10-year cohort analysis of routine paediatric ART data in a rural South African setting

**DOI:** 10.1017/S0950268816001916

**Published:** 2016-09-09

**Authors:** R. R. LILIAN, B. MUTASA, J. RAILTON, W. MONGWE, J. A. McINTYRE, H. E. STRUTHERS, R. P. H. PETERS

**Affiliations:** 1 Anova Health Institute, Johannesburg and Tzaneen, South Africa; 2 Mopani Department of Health, Giyani, South Africa; 3 School of Public Health and Family Medicine, University of Cape Town, Cape Town, South Africa; 4 Department of Medicine, University of Cape Town, Cape Town, South Africa; 5 Department of Microbiology, University of Pretoria, Pretoria, South Africa

**Keywords:** Analysis of data, HIV/AIDS, paediatrics, public health

## Abstract

South Africa's paediatric antiretroviral therapy (ART) programme is managed using a monitoring and evaluation tool known as TIER.Net. This electronic system has several advantages over paper-based systems, allowing profiling of the paediatric ART programme over time. We analysed anonymized TIER.Net data for HIV-infected children aged <15 years who had initiated ART in a rural district of South Africa between 2005 and 2014. We performed Kaplan–Meier survival analysis to assess outcomes over time. Records of 5461 children were available for analysis; 3593 (66%) children were retained in care. Losses from the programme were higher in children initiated on treatment in more recent years (*P* < 0·0001) and in children aged ≤1 year at treatment initiation (*P* < 0·0001). For children aged <3 years, abacavir was associated with a significantly higher rate of loss from the programme compared to stavudine (hazard ratio 1·9, *P* < 0·001). Viral load was suppressed in 48–52% of the cohort, with no significant change over the years (*P* = 0·398). Analysis of TIER.Net data over time provides enhanced insights into the performance of the paediatric ART programme and highlights interventions to improve programme performance.

## INTRODUCTION

An estimated 340 000 children aged <15 years are living with human immunodeficiency virus (HIV) in South Africa [1]. About 167 000 (49%) of these children receive antiretroviral therapy (ART) [1], representing the largest paediatric HIV treatment programme in the world. HIV-infected children are at significant risk of excess morbidity and mortality [2, 3] and it is therefore essential that the ART programme be effectively managed to ensure high-quality care for these children.

The ART programme in the South African public healthcare sector has undergone a number of changes to accommodate the large number of adult and paediatric HIV patients. A key policy change has been programme expansion through decentralization of ART management to primary health centres, employing nurse-managed, as opposed to doctor-managed, models [4]. It has been shown that nurse-monitored ART delivery in adult patients is non-inferior to doctor-monitored treatment [5], with Nurse Initiated Management of ART (NIMART) increasing ART uptake and reducing workload at referral facilities [6]. Task-shifting of ART initiation and management to non-physicians for paediatric patients has also been shown to provide comparable clinical and programme outcomes [7]. Expansion of South Africa's ART programme further encompassed changes to treatment eligibility criteria. When the programme was launched in 2004, immunological and clinical criteria determined ART eligibility in children [8]. In 2010, all infants aged <1 year became eligible for ART, irrespective of CD4 count or clinical stage [9], and in August 2012 all children aged <5 years became eligible for treatment [10]. First-line regimens were simultaneously adapted. In 2004, children aged <3 years were initiated on stavudine, lamivudine and lopinavir/ritonavir (S3L) while older children received stavudine, lamivudine and efavirenz (S3E) [8]. In 2010, stavudine was replaced with abacavir in all children experiencing side-effects and recommended first-line regimens were abacavir, lamivudine and lopinavir/ritonavir (A3L) in children aged <3 years and abacavir, lamivudine and efavirenz (A3E) in children aged >3 years [9]. From 2013, all children with undetectable viral loads (VL) who had been initiated on stavudine were also switched to abacavir [11].

Expansion of the ART programme has necessitated an efficient monitoring and evaluation (M&E) system to manage the increasing number of children on ART. In December 2010, the South African National Department of Health adopted an ART M&E tool known as TIER.Net (Three Interlinked Electronic Registers.Net) which was developed by the University of Cape Town's Centre for Infectious Disease Epidemiology and Research [12]. TIER.Net is a three-phase system, progressing from paper registers (tier 1) to stand-alone electronic registers (tier 2) and finally to networked electronic medical records (tier 3) [12]. The majority of facilities have implemented tier 2 and all historical ART data have been retrospectively captured. TIER.Net is used operationally to monitor baseline clinical care and patient outcomes over time, facilitating tracing of patients who have missed appointments or defaulted from care. Routine data captured electronically in TIER.Net provide a rich source of information and allow for detailed analysis of programme performance over time. Such analyses are important for understanding temporal changes in the performance of the ART programme, demonstrating associations between programme expansion and patient outcomes, and also provide opportunities for comparing cohorts over time and between time periods. These insights are essential to improve the long-term effectiveness of the ART programme and are particularly important as the programme is expanded to achieve 90-90-90 targets [13]. Electronic TIER.Net data are readily available for the paediatric ART programme and provide a valuable opportunity to assess programme performance over time, which to our knowledge has not previously been performed. In the present study, to highlight the importance of the availability of such data, we present an analysis of the paediatric ART programme using routine TIER.Net data for children initiating ART over a 10-year period in a rural South African district.

## METHODS

### Data source and study design

We analysed routinely available, anonymized TIER.Net data for children initiating ART in Mopani district of Limpopo Province, South Africa, which has an antenatal HIV prevalence of 24·6% [14]. Paediatric TIER.Net tier 2 data, extracted from TIER.Net in February 2015, were available for 106/109 (97%) facilities offering ART services. The following criteria were used to select records for inclusion in the study: child aged <15 years at ART initiation, ART initiation between January 2005 and December 2014 and documentation of key dates in TIER.Net (date of birth, ART initiation date and date of last ART visit). Records were excluded where tenofovir disoproxil fumarate (TDF) had been captured in the ART regimen in order to avoid misclassification of adults as children through incorrect capturing of birth dates, as this drug is only indicated for individuals aged ⩾15 years according to South African guidelines [15]. Records were also excluded where children transferred out of Mopani's ART programme.

### Ethical approval

The study was approved by the University of the Witwatersrand's Medical Ethics Committee (clearance number M140461) and the Limpopo Provincial Health Research Committee of the Department of Health. We analysed anonymized TIER.Net data that were routinely collected at healthcare facilities for monitoring purposes and individual consent was therefore not required. No patient files or electronic medical records were accessed at any stage.

### Definitions of programme and virological outcomes

We classified programme outcomes as in care, dead or lost to follow-up (LTFU). Children who had a last recorded visit within 120 days of the facility's last data update were classified as in care. Children who died were designated as such in the original TIER.Net extract. LTFU was defined as a last recorded ART visit >120 days before the facility data were last updated. A definition of 120 days from the last ART visit equates to 90 days without drug in hand, in line with the definition of LTFU in TIER.Net, as children would have received a 30-day supply of medication at the last ART visit. Follow-up time was defined as the time between the date of ART initiation and date of last ART visit. Virological outcomes were classified using recent VL results, defined as tests that were performed within 1 year of the facility's last data update. Viral suppression was defined as a last VL result <400 copies/ml.

### Statistical analysis

Where ART regimens were analysed, only valid regimens as per South African guidelines that were captured correctly in TIER.Net were included in the analysis. ART initiation was divided into three periods (initiations prior to April 2010, between April 2010 and August 2012 and after August 2012) to reflect the 2010 and 2012 ART eligibility guideline changes [9, 10]. Cohort characteristics were compared across these periods using Kruskal–Wallis analysis of variance and *χ*^2^ or Fisher's exact tests for continuous and categorical variables, respectively. *Post-hoc* testing was performed using Mann–Whitney *U, χ*^2^ and Fisher's exact tests as appropriate. Virological outcomes for children in care as at December 2014 who had received treatment for at least 6 months were stratified over the ART initiation periods and similarly analysed. *P* < 0·05 was considered significant.

Kaplan–Meier survival analysis was used to estimate the probability of death or LTFU over time. Follow-up time was censored at 5 years after ART initiation. For children who were LTFU after their initiation visit (i.e. ART initiation date and last visit date were the same), a follow-up time of half a day (0·001 years) was assigned. Survival curves were compared using a Log-rank test. Cox proportional hazard models were used to determine characteristics associated with loss from the ART programme. All analyses were performed using Stata v. 13.0 (StataCorp LP, USA).

## RESULTS

### Description of study population

The dataset from TIER.Net comprised 7206 records of children who had initiated ART in Mopani. Records from 1745 (24%) children were excluded for not meeting inclusion criteria, including 1345 children who had transferred out of the ART programme and 400 with overlapping data quality problems, including ART regimens in which TDF had been captured (*n* = 383), inaccurate ART initiation dates from years when the ART programme had not been initiated (*n* = 18) and a missing last ART visit date (*n* = 1), leaving 5461 records for analysis. A higher proportion of children who transferred out of the programme had initiated ART prior to 2012 compared to those included in the analysis (*P* < 0·001); these children therefore had lower baseline CD4 counts (*P* < 0·0001) and were more likely to have been initiated on a stavudine-based regimen at baseline (*P* < 0·001) (see Supplementary Table S1). Of the 5461 children included in the analysis, 5331 (97·6%) and 5457 (99·9%) had baseline and last ART regimens recorded, respectively, of which <1% of the captured regimens were invalid (*n* = 11 and 50, respectively).

**Fig. 1.**
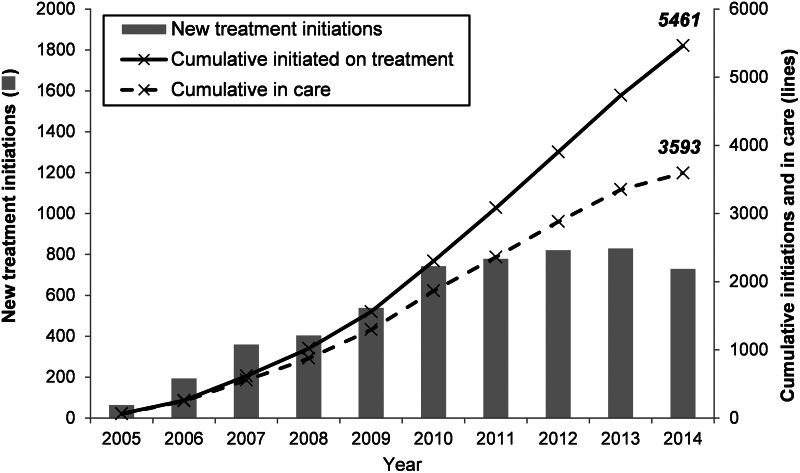
Growth of Mopani's paediatric antiretroviral treatment programme over time.

The paediatric ART programme in Mopani has expanded over time, with new ART initiations increasing steadily from 2005 to 2011, followed by a marginal decrease in 2014 (Fig. 1). One third (34·2%) of the 5461 children who had been initiated on ART between 2005 and 2014 died or were LTFU (*n* = 300 and 1568, respectively), leaving a cumulative total of 3593 children in care at the end of 2014.

Median follow-up time of the 5461 children in the cohort was 2·2 years (range 0·001–9·8 years). Even though the latter two ART initiation periods spanned ~2 years compared to the first period which spanned >5 years, comparable numbers of children were initiated on treatment in these periods as a result of expansion of the ART programme in the latter years (Table 1). Median age at initiation decreased (*P* = 0·0001) and baseline CD4 count increased (*P* = 0·0001) over the three ART initiation periods. In children who were aged ≤3 years at ART initiation, the proportion receiving abacavir increased over time, while stavudine decreased as per national ART guidelines (*P* < 0·001). The same trend was evident in children aged >3 years at ART initiation (*P* < 0·001).

**Table 1.** Cohort characteristics at treatment initiation by ART initiation period

	1. ART initiations prior to April 2010	2. ART initiations between April 2010 and August 2012	3. ART initiations after August 2012	*P*
*N*	1720	1897	1844	
Gender, *n* (%)				0·323
Male	830 (48·3%)	960 (50·6%)	899 (48·8%)	
Female	890 (51·7%)	937 (49·4%)	945 (51·3%)	
Age at initiation, years, median (range)	5·3 (0·001–15·0)	5·2 (0·008–15·0)	4·6 (0·005–15·0)	0·0001*
On TB treatment at ART initiation, *n* (%)				**<0·001†**
Yes	74 (5·3%)	109 (6·7%)	165 (10·2%)	
No	1333 (94·7%)	1527 (93·3%)	1460 (89·9%)	
Baseline CD4 count cells/mm^3^, median (range)	160·5 (0–2506)	236 (2–3000)	325 (1–2768)	**0·0001**‡
Baseline regimen in children aged ⩽3 years at ART initiation, *n* (%)				**<0·001**§
A3L	27 (12·9%)	390 (83·5%)	655 (99·5%)	
S3L	183 (87·1%)	77 (16·5%)	3 (0·5%)	
Baseline regimen in children aged >3 years at ART initiation, *n* (%)				**<0·001**§
A3E	55 (5·7%)	596 (58·8%)	882 (92·7%)	
S3E	916 (94·3%)	417 (41·2%)	69 (7·3%)	

A3E, Abacavir, lamivudine and efavirenz; A3L, abacavir, lamivudine and lopinavir; ART, antiretroviral therapy; S3E, stavudine, lamivudine and efavirenz; S3L, stavudine, lamivudine and lopinavir; TB, tuberculosis.

Statistically significant differences are shown in bold.

* Significant difference between ART initiation periods 2 and 3 (*P* < 0·001) and 1 and 3 (*P* = 0·0001).

† Significant difference between ART initiation periods 2 and 3 (*P* < 0·001) and 1 and 3 (*P* < 0·001).

‡ Significant difference between ART initiation periods 1 and 2 (*P* < 0·001), 1 and 3 (*P* < 0·0001) and 2 and 3 (*P* < 0·0001).

§ Significant difference between ART initiation periods 1 and 2 (*P* < 0·001), 1 and 3 (*P* < 0·001) and 2 and 3 (*P* < 0·001).

### Programme outcomes over time

**Fig. 2.**
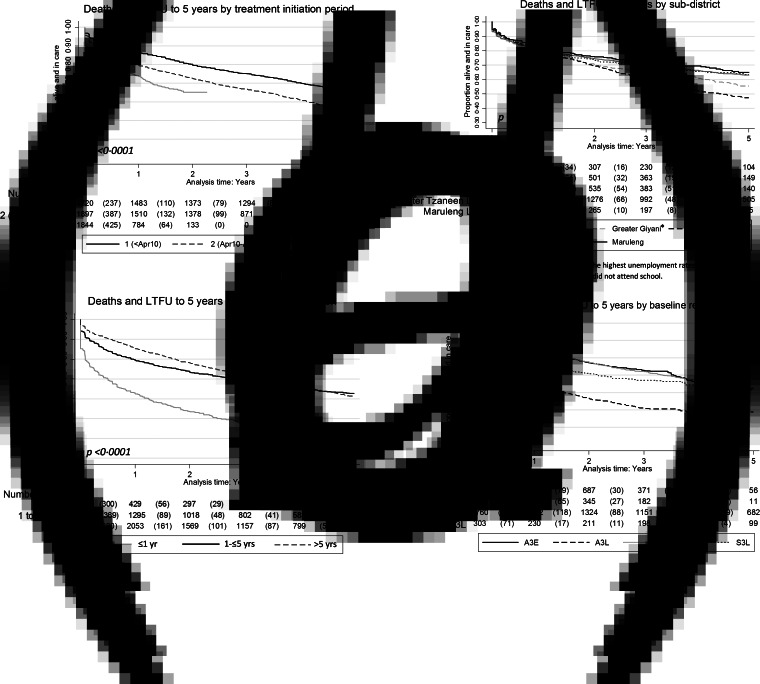
Retention in care to 5 years on treatment by (*a*) treatment initiation period, (*b*) subdistrict, (*c*) age at treatment initiation and (*d*) baseline regimen (*P* = log-rank test). A3E, Abacavir, lamivudine and efavirenz; A3L, abacavir, lamivudine and lopinavir; ART, antiretroviral therapy; LTFU, losses to follow-up; S3E, stavudine, lamivudine and efavirenz; S3L, stavudine, lamivudine and lopinavir.

Retention over time was equal in male and female children, with 20% LTFU or reported dead by 1 year on treatment (*n* = 519 males, 530 females) and 41% lost by 5 years (*n* = 35 and *n* = 48, respectively) (*P* = 0·717). Children initiated on ART in the most recent ART initiation period were lost from the programme more rapidly than those initiated in the early years, with 1-year losses of 26% in children initiated after August 2012 compared to 14% in children initiated prior to April 2010 [hazard ratio (HR) 2·0, *P* < 0·001] (Fig. 2*a*). This trend was more marked in infants aged ≤1 year at ART initiation, with 15% more infants lost by 1 year in those initiated after August 2012 compared to early initiations prior to April 2010, as opposed to a difference of only 8% in children aged >5 years at ART initiation (see Supplementary Fig. S1*a*). Losses from the programme were higher in the two subdistricts in Mopani with the lowest education and employment rates (Greater Letaba and Greater Giyani [16]; *P* < 0·0001) (Fig. 2*b*) and in younger children aged ≤1 year at treatment initiation (*P* < 0·0001) (Fig. 2*c*). In older children on efavirenz, there was no difference in retention between abacavir (A3E) and stavudine (S3E) (HR 1·0, *P* = 0·465), but in younger children on lopinavir, abacavir (A3L) was associated with a significantly higher rate of loss from the programme compared to stavudine (S3L) (HR 1·9, *P* < 0·001) (Fig. 2*d*). This trend was still significant when early losses at 0·001 years (*n* = 386) were excluded from the analysis (HR 1·8 for A3L compared to S3L, *P* < 0·001) (see Supplementary Fig. S1*b*).

### Virological outcomes

The proportion of children in care who had a recent VL result recorded remained relatively steady over time, with a slight decrease in children initiated later in the programme (44·7%, 45·5% and 40·5% in children initiated in periods 1, 2 and 3, respectively; *P* = 0·042). VL testing did not differ in children on abacavir- *vs.* stavudine-based regimens (*P* = 1·000 and 0·246 for children aged under and over 3 years, respectively). The size of the facility attended at the last ART visit was consistently associated with VL testing across all ART initiation periods, with a higher proportion of children who did not access VL testing coming from larger facilities (*P* = 0·001, <0·001 and 0·006 in periods 1, 2 and 3, respectively) (Table 2). No other factor was consistently associated with VL testing. In the 1433 children with a recent VL recorded, median VL was higher in children ⩽3 *vs.* >3 years at the time of VL testing (3·3 *vs.* 2·6 log, respectively; *P* = 0·0001).

**Table 2.** Characteristics of children in care by viral load testing status and ART initiation period

	Recent viral load	No recent viral load	*P*
**ART initiations prior to April 2010, *n***	456	565	
Facility catchment population, *n* (%)			0·001*
≤5000	9 (3·3%)	23 (4·6%)	
5000–10 000	123 (45·4%)	159 (31·6%)	
>10 000	139 (51·3%)	321 (63·8%)	
Gender, *n* (%)			0·909
Male	226 (49·6%)	278 (49·2%)	
Female	230 (50·4%)	287 (50·8%)	
Time on treatment, years, median (range)	6·3 (4·7–9·9)	6·1 (4·7–9·9)	0·514
Current age, years, median (range)	11·7 (5·2–22·8)	11·9 (4·9–22·9)	0·419
**ART initiations between April 2010 and August 2012, *n***	554	663	
Facility catchment population, *n* (%)			**<0·001**†
≤5000	26 (6·9%)	24 (4·1%)	
5000–10 000	209 (55·7%)	219 (37·4%)	
>10 000	140 (37·3%)	342 (58·5%)	
Gender, *n* (%)			**0·041**
Male	295 (53·3%)	314 (47·4%)	
Female	259 (46·8%)	349 (52·6%)	
Time on treatment, years, median (range)	3·4 (2·3–4·7)	3·5 (2·3–4·7)	0·063
Current age, years, median (range)	9·4 (2·6–19·4)	8·8 (2·6–18·5)	0·109
**ART initiations after August 2012, *n***	423	621	
Facility catchment population, *n* (%)			**0·006**‡
≤5000	20 (6·3%)	16 (2·8%)	
5000–10 000	159 (50·2%)	263 (45·5%)	
>10 000	138 (43·5%)	299 (51·7%)	
Gender, *n* (%)			0·804
Male	199 (47·0%)	297 (47·8%)	
Female	224 (53·0%)	324 (52·2%)	
Time on treatment, years, median (range)	1·3 (0·5–2·3)	1·4 (0·5–2·3)	0·573
Current age, years, median (range)	7·4 (0·7–16·8)	6·7 (0·6–17·0)	0·093

ART, Antiretroviral therapy.

Statistically significant differences are shown in bold.

* Significant difference between the 5000–10 000 and >10 000 groups (*P* < 0·001).

† Significant difference between the 5000–10 000 and >10 000 groups (*P* < 0·001) and the ≤5000 and >10 000 groups (*P* = 0·001).

‡ Significant difference between the ≤5000 and >10 000 groups (*P* = 0·003) and the ≤5000 and 5000–10 000 groups (*P* = 0·035).

In children in care with a recent VL result, viral suppression was documented in about half of the cohort (51·5%, 47·8% and 47·5% in ART initiation periods 1, 2 and 3, respectively; *P* = 0·398). Suppression did not differ by gender (*P* = 0·656). Compared to children who were not virally suppressed, children with a suppressed VL started ART at younger ages (*P* < 0·001 and <0·0001 in periods 1 and 2, respectively) and as expected, had significantly higher CD4 counts (*P* < 0·0001, <0·0001 and 0·013 in periods 1, 2 and 3, respectively) (Table 3). By Kaplan–Meier analysis, children who were not suppressed were significantly more likely to be lost from the programme compared to children who were virally suppressed, although the absolute hazard was low (1-year losses of 3·5% *vs.* 0·1%, respectively; HR 6·0, *P* < 0·001; see Supplementary Fig. S1*c*).

**Table 3.** Characteristics of children in care with a recent viral load result by suppression status and ART initiation period

	Suppressed	Not suppressed	*P*
**ART initiations prior to April 2010, *n***	235	221	
Age at ART initiation, years, median (range)	4·4 (0·001–14·2)	5·6 (0·2–14·8)	**<0·001**
Time on treatment, years, median (range)	6·2 (4·7–9·9)	6·3 (4·7–9·7)	0·380
Last CD4 count, cells/mm^3^, median (range)	771 (24–2139)	543 (3–2242)	**<0·0001**
ART regimen, *n* (%)			**0·023**
First line	200 (97·6%)	148 (92·5%)	
Second line*	5 (2·4%)	12 (7·5%)	
Last ART regimen in children aged >3 years, *n* (%)			**<0·001**
A3E	148 (83·2%)	83 (63·9%)	
S3E	30 (16·9%)	47 (36·2%)	
**ART initiations between April 2010 and August 2012, *n***	265	289	
Age at ART initiation, years, median (range)	4·9 (0·2–14·8)	7·1 (0·1–15·0)	**<0·0001**
Time on treatment, years, median (range)	3·5 (2·3–4·7)	3·3 (2·3–4·7)	0·321
Last CD4 count, cells/mm^3^, median (range)	784 (12–2777)	506·5 (0–2899)	**<0·0001**
ART regimen, *n* (%)			0·062
First line	237 (98·3%)	250 (95·4%)	
Second line†	4 (1·7%)	12 (4·6%)	
Last ART regimen in children aged ⩽3 years, *n* (%)			—
A3L	5 (100%)	3 (100%)	
S3L	0 (0·0%)	0 (0·0%)	
Last ART regimen in children aged >3 years, *n* (%)			0·081
A3E	154 (91·7%)	163 (85·8%)	
S3E	14 (8·3%)	27 (14·2%)	
**ART initiations after August 2012, *n***	201	222	
Age at ART initiation, years, median (range)	5·7 (0·1–14·6)	6·2 (0·1–14·9)	0·896
Time on treatment, years, median (range)	1·5 (0·5–2·3)	1·2 (0·5–2·3)	**0·0001**
Last CD4 count, cells/mm^3^, median (range)	793 (21–2989)	486 (9–2895)	**0·013**
ART regimen, *n* (%)			0·250
First line	196 (100%)	214 (98·6%)	
Second line‡	0 (0·0%)	3 (1·4%)	
Last ART regimen in children aged ⩽3 years, *n* (%)			—
A3L	27 (100%)	51 (100%)	
S3L	0 (0·0%)	0 (0·0%)	
Last ART regimen in children aged >3 years, *n* (%)			0·076
A3E	133 (97·8%)	127 (93·4%)	
S3E	3 (2·2%)	9 (6·6%)	

A3E, Abacavir, lamivudine and efavirenz; A3L, abacavir, lamivudine and lopinavir; ART, antiretroviral therapy; S3E, stavudine, lamivudine and efavirenz; S3L, stavudine, lamivudine and lopinavir.

Statistically significant differences are shown in bold.

* The switch to second line regimens occurred a median 4·8 years after ART initiation.

† The switch to second line regimens occurred a median 2·6 years after ART initiation.

‡ The switch to second line regimens occurred a median 1·4 years after ART initiation.

## DISCUSSION

This study documents expansion of the paediatric ART programme over time in a rural South African district, with children initiated on treatment at increasingly younger ages and higher baseline CD4 counts in line with changing guidelines [8–11]. In recent years, the number of children initiated on treatment per annum has plateaued and somewhat declined, likely a result of reduced infant infections due to successes in the prevention of mother-to-child transmission programme [17]. We show that 20% of children were LTFU or reported dead by 1 year on treatment, consistent with the 16% 1-year failure estimate at a tertiary children's hospital in South Africa [18], but higher than the 1-year attrition rates of 8–12% in other multi-centre South African cohorts of children aged <16 years [19, 20]. These differences may be attributed to differing time periods with varying ART guidelines, a higher proportion of younger children in our cohort [20] and different definitions of LTFU, with one study using a notably longer period of 6 months [19]. Attrition through the paediatric ART programme is a known problem [20–22], with increased rates of mortality and LTFU soon after initiating treatment as was also noted in this study [19, 22]. Interventions to curb attrition are urgently required and may include community adherence support programmes [20] and ensuring timely ART initiation, as severe clinical decline increases the hazard of death and LTFU [21].

The mortality rate of children classified as LTFU in our cohort is not known, although rates of 33–39% have been reported in other paediatric cohorts [20, 23]. To minimize the impact of unreported deaths, deaths and LTFU were analysed together in survival analyses and a concerning trend of decreasing rates of retention in recent years of the paediatric ART programme was demonstrated. This trend has been noted in multiple studies of adult patients, with LTFU occurring earlier and at higher rates in patients initiated in successive years of the ART programme [24–27]. Although paediatric studies have demonstrated reduced mortality in children initiating treatment in later years [19, 22], rates of LTFU have been shown to be increasing over time [19, 28]. A single study found a trend of reduced LTFU in recent years, but this was not significant and the study was only performed in infants aged <1 year at ART initiation [22]. The trend of decreasing rates of retention may be a result of the increasing number of patients in the ART programme [26], as rate of programme expansion is strongly associated with increased LTFU [29], or more specifically, due to the treatment of increasing numbers of healthy patients. Effectively managing the increasing numbers of patients in the ART programme requires expanded resources, training and decongestion of ART services by moving chronic, stable patients to separate programmes. Additionally, it is important to consider social factors, as socioeconomic deficiencies have been shown to increase the risk of both death and LTFU [27, 30, 31] in agreement with findings in our study.

Our study further demonstrates increased risk of attrition in children aged ≤1 year at ART initiation and higher VL in younger children. Many previous studies have documented increased mortality and LTFU in younger children, particularly in those younger than 1 or 2 years [19, 20, 28, 32]. The functionally immature immune system of infants leaves them susceptible to viral and bacterial infections [33], while reliance on caregivers and the need for frequent administration of often unpalatable medications complicates paediatric care [34]. Lopinavir/ritonavir in particular is known to be challenging due to poor palatability [34], potentially increasing the risk of poor outcomes in young children who receive lopinavir-based regimens. In particular, we found that young children receiving abacavir (A3L) had a significantly higher rate of attrition compared to those receiving stavudine (S3L), which is particularly problematical in view of the proportionately increasing number of children receiving A3L as per national guidelines [9, 11]. This is a complex matter that warrants further research, particularly since these findings are consistent with observations by another South African research group that reported poor virological outcomes in children receiving abacavir, with proportionally fewer children reaching suppression and shorter time to viral rebound compared to those receiving stavudine [35, 36]. These differences may be attributable to guideline and programmatic changes over time, as abacavir-based regimens were introduced after stavudine [35, 36]. Nevertheless, pharmacological characteristics [35, 37] and treatment interruptions due to abacavir stock-outs [35, 36] may also have contributed to poor outcomes in children receiving A3L. In our setting, stock-outs of abacavir syrup have also been reported and poor supply-chain management may therefore have contributed to treatment interruptions and ultimately poor adherence in children initiated on A3L.

Of further concern is the low rate of VL testing in our cohort, with only 40–45% of children in care having a recent VL result on record and no improvement in this rate over time. Failure to capture VL results in TIER.Net and lack of confidence among nursing staff in managing paediatric patients may have contributed to the apparently low testing rate in our cohort. It would be of interest in future studies to link patient-level TIER.Net data to laboratory VL records to ascertain the relative contributions of data capturing problems *vs.* lapses in clinical care. Other paediatric studies in South Africa have documented higher VL testing rates of 60–80% [19, 36] but these studies included only urban sites where laboratory testing and skilled staff may have been more readily available than in the rural setting in our study. Even testing rates of 60–80% are a concern in light of the 90-90-90 targets which aim to achieve viral suppression in 90% of children on treatment [13], thus necessitating VL testing in virtually all children on ART. Of interest, we found that implementation of VL testing is particularly poor at facilities with large catchment populations, in line with findings that considerably fewer patients at district or regional hospitals have available VL results compared to primary healthcare facilities [26]. This may be due to operational challenges that face high-throughput facilities, with a considerable burden on staff, infrastructure and resources.

Viral suppression rates in our cohort were also low, with only half the children in care with a recent VL result having achieved suppression. Reported suppression rates in South African paediatric cohorts range from 56% to 82% in children with VL results [18–20, 22], equating to 27% of HIV-infected children aged <15 years [38]. The low rate of suppression is concerning in light of the short- and long-term clinical implications and the increased risk of loss from the ART programme in children who are not suppressed. Children should be initiated on treatment at younger ages in order to improve suppression rates, as viral suppression is associated with younger baseline age in this and other studies [36]. This reinforces calls for early diagnosis and early ART initiation to reduce paediatric morbidity and mortality [39]. Furthermore, systems to flag high VL results are needed and adherence, crucial to achieving and maintaining viral suppression, must be reinforced – ongoing counselling must be provided from the time of ART initiation to address children's changing adherence barriers, and community-based adherence support should be considered with community workers providing education and psychosocial support to address household challenges impacting on adherence [40, 41].

To our knowledge, this is the first in-depth analysis of a South African district's paediatric ART programme over a 10-year period using routine TIER.Net data. Analysing data from TIER.Net has several advantages, including access to standardized data from a substantial number of children from multiple sites. In this study, the risk of double-counting was minimized by extracting data at a single point in time. In addition, the analysis was performed using data from a number of years in which there were substantial guideline changes in the South African ART programme, providing a realistic and robust analysis. On the other hand, this study has several limitations: data quality was a challenge and a number of records had to be excluded because of a missing last ART visit date, inaccurate ART initiation dates from years when the ART programme had not been initiated or ART regimens in which TDF had been captured. The latter raised concerns regarding the accuracy of the recorded dates of birth, as TDF is only given to adult patients. Programme interventions to improve the quality of data captured in TIER.Net are essential. In addition, excluding children who transferred out of Mopani's ART programme from the analysis may have biased our findings to children who were initiated later in the ART programme, as a higher proportion of these children had initiated ART prior to 2012. Finally, these findings from a rural South African district should be generalized to urban settings and other countries with caution.

In conclusion, routine data captured electronically in TIER.Net allows multi-site programme analysis that would be difficult to perform using paper-based registers. Analysis of these data from a rural district in South Africa demonstrates substantial growth of the paediatric ART programme over time. However, challenges remain with regard to virological testing, suppression rates and retention in care, particularly in children living in poorer socioeconomic areas, infants and children aged <3 years receiving abacavir-based regimens. These children need to be targeted for improved care, and programme planning and implementation, including supply chain management, needs to be enhanced if paediatric outcomes are to be improved. These findings demonstrate the value of TIER.Net data in providing enhanced insights into the performance of the paediatric ART programme, highlighting interventions to improve the long-term effectiveness of the programme.

## Supplementary material

For supplementary material accompanying this paper visit http://doi.org/10.1017/S0950268816001916. click here to view supplementary material
