# Supplementary material for: A 10-year cohort analysis of routine paediatric ART data in a rural South African setting
Source: Epidemiol Infect. 2016 Sep 9;145(1):170–80. doi: 10.1017/S0950268816001916 (PMC5197927; doi:10.1017/S0950268816001916)
Supplement: Supplementary file 1 [file S0950268816001916sup.zip › S0950268816001916sup001.docx]

Supplementary Table S1. Adjusted hazard ratios (95% confidence intervals) for the association of trauma exposure and PTSD symptoms with risk of incident hypertension, 1989-2011, presented for different reference groups.

| **Reference Group: Trauma/No Symptoms** | | | | | | | | | | |
| --- | --- | --- | --- | --- | --- | --- | --- | --- | --- | --- |
|  |  |  | Trauma-exposed | | | | | | | |
|  | No trauma | | No symptoms | | 1-3 symptoms | | 4-5 symptoms | | 6-7 symptoms | |
|  | HR (95% CI) | *P* | HR | | HR (95% CI) | *P* | HR (95% CI) | *P* | HR (95% CI) | *P* |
| *Model 1** | 0.96 (0.92-0.998) | .038 | 1 (ref) | | 1.08 (1.02-1.13) | .003 | 1.12 (1.06-1.19) | <.0001 | 1.15 (1.08-1.24) | <.0001 |
| *Model 2*^+^ | 0.98 (0.94-1.02) | .276 | 1 (ref) | | 1.07 (1.02-1.12) | .008 | 1.10 (1.04-1.16) | .001 | 1.10 (1.03-1.18) | .006 |
| *Model 3*^†^ | 1.00 (0.96-1.04) | .919 | 1 (ref) | | 1.07 (1.02-1.12) | .010 | 1.09 (1.03-1.16) | .003 | 1.08 (1.005-1.16) | .036 |
| *Model 4*^§^ | 1.00 (0.96-1.04) | .979 | 1 (ref) | | 1.06 (1.01-1.11) | .018 | 1.08 (1.02-1.14) | .012 | 1.06 (0.99-1.13) | .122 |
| **Reference Group: Trauma/1-3 Symptoms** | | | | | | | | | | |
|  | | | Trauma-exposed | | | | | | | |
|  | No trauma | | No symptoms | | 1-3 symptoms | | 4-5 symptoms | | 6-7 symptoms | |
|  | HR (95% CI) | *P* | HR (95% CI) | *P* | HR | | HR (95% CI) | *P* | HR (95% CI) | *P* |
| *Model 1** | 0.89 (0.85-0.94) | <.0001 | 0.93 (0.89-0.98) | .003 | 1 (ref) | | 1.04 (0.98-1.12) | .210 | 1.07 (0.99-1.16) | .073 |
| *Model 2*^+^ | 0.92 (0.87-0.97) | .002 | 0.94 (0.89-0.98) | .008 | 1 (ref) | | 1.03 (0.96-1.10) | .379 | 1.03 (0.96-1.12) | .411 |
| *Model 3*^†^ | 0.94 (0.89-0.99) | .018 | 0.94 (0.89-0.98) | .010 | 1 (ref) | | 1.02 (0.96-1.10) | .501 | 1.01 (0.94-1.09) | .777 |
| *Model 4*^§^ | 0.94 (0.89-0.995) | .034 | 0.94 (0.90-0.99) | .018 | 1 (ref) | | 1.02 (0.95-1.09) | .641 | 1.00 (0.92-1.08) | .948 |
| **Reference Group: Trauma/4-5 Symptoms** | | | | | | | | | | |
|  |  |  | Trauma-exposed | | | | | | | |
|  | No trauma | | No symptoms | | 1-3 symptoms | | 4-5 symptoms | | 6-7 symptoms | |
|  | HR (95% CI) | *P* | HR (95% CI) | *P* | HR (95% CI) | *P* | HR | | HR (95% CI) | *P* |
| *Model 1** | 0.85 (0.80-0.91) | <.0001 | 0.89 (0.84-0.94) | <.0001 | 0.96 (0.90-1.02) | .210 | 1 (ref) | | 1.03 (0.95-1.12) | .513 |
| *Model 2*^+^ | 0.89 (0.84-0.95) | .0002 | 0.91 (0.86-0.96) | .001 | 0.97 (0.91-1.04) | .379 | 1 (ref) | | 1.00 (0.92-1.09) | .955 |
| *Model 3*^†^ | 0.92 (0.86-0.97) | .006 | 0.92 (0.87-0.97) | .003 | 0.98 (0.91-1.05) | .501 | 1 (ref) | | 0.99 (0.91-1.08) | .781 |
| *Model 4*^§^ | 0.93 (0.87-0.99) | .020 | 0.93 (0.88-0.98) | .012 | 0.98 (0.92-1.05) | .641 | 1 (ref) | | 0.98 (0.90-1.07) | .663 |
| **Reference Group: Trauma/6-7 Symptoms** | | | | | | | | | | |
|  |  |  | Trauma-exposed | | | | | | | |
|  | No trauma | | No symptoms | | 1-3 symptoms | | 4-5 symptoms | | 6-7 symptoms | |
|  | HR (95% CI) | *P* | HR (95% CI) | *P* | HR (95% CI) | *P* | HR (95% CI) | *P* | HR | |
| *Model 1** | 0.83 (0.77-0.89) | <.0001 | 0.87 (0.81-0.93) | <.0001 | 0.93 (0.86-1.01) | .073 | 0.97 (0.89-1.06) | .513 | 1 (ref) | |
| *Model 2*^+^ | 0.89 (0.82-0.95) | .001 | 0.91 (0.85-0.97) | .006 | 0.97 (0.90-1.05) | .411 | 1.00 (0.92-1.09) | .955 | 1 (ref) | |
| *Model 3*^†^ | 0.93 (0.86-0.998) | .043 | 0.93 (0.87-0.995) | .036 | 0.99 (0.91-1.07) | .777 | 1.01 (0.93-1.10) | .781 | 1 (ref) | |
| *Model 4*^§^ | 0.95 (0.88-1.02) | .142 | 0.95 (0.88-1.01) | .122 | 1.00 (0.93-1.08) | .948 | 1.02 (0.94-1.11) | .663 | 1 (ref) | |

*Note*. HR=hazard ratio. 95% CI=95% confidence interval.

*Adjusted for age at baseline, race/ethnicity, parental education, maternal and paternal history of hypertension, and age 5 somatotype.

^+^Additionally adjusted for use of oral contraceptives, acetaminophen, aspirin, and other nonsteroidal anti-inflammatory drugs, menopausal status and hormone therapy use, and hypercholesterolemia.

^†^Additionally adjusted for body mass index, physical activity, diet quality, sugar-sweetened beverage consumption, artificially-sweetened beverage consumption, cigarette smoking, and alcohol consumption.

^§^Additionally adjusted for antidepressant use.

Supplementary Table S2. Adjusted hazard ratios (95% confidence intervals) for the variables in Model 2 with risk of incident hypertension, 1989-2011

| Predictor | Hazard Ratio (95% Confidence Interval) | | | *P* |
| --- | --- | --- | --- | --- |
| Trauma/PTSD symptom status |  | | |  |
| No trauma | 1 (ref) | | | --- |
| Trauma/no symptoms | 1.02 (0.98-1.06) | | | .283 |
| Trauma/1-3 symptoms | 1.09 (1.03-1.15) | | | .002 |
| Trauma/4-5 symptoms | 1.13 (1.06-1.20) | | | .0002 |
| Trauma/6-7 symptoms | 1.13 (1.05-1.21) | | | .002 |
| Parents’ education at birth |  | | |  |
| High school or less | 1 (ref) | | | --- |
| Some college | 0.96 (0.92-1.00) | | | .029 |
| College or more | 0.85 (0.81-0.88) | | | <.0001 |
| Missing | 1.00 (0.94-1.06) | | | .973 |
| Maternal history of hypertension | 1.57 (1.52-1.62) | | | <.0001 |
| Paternal history of hypertension | 1.45 (1.41-1.50) | | | <.0001 |
| Race/ethnicity |  | | |  |
| White | 1 (ref) | | | --- |
| African American | 1.69 (1.48-1.94) | | | <.0001 |
| Latina | 0.92 (0.80-1.07) | | | .271 |
| Asian | 1.21 (1.06-1.38) | | | .005 |
| Other | 0.97 (0.85-1.11) | | | .646 |
| Missing | 0.88 (0.75-1.02) | | | .080 |
| Somatotype at age 5 |  |  |  |  |
| Category 1 (smallest) | 1.03 (0.99-1.07) | | | .195 |
| Category 2 | 1 (ref) | | | --- |
| Category 3 | 1.08 (1.03-1.12) | | | .001 |
| Category 4 | 1.17 (1.11-1.23) | | | <.0001 |
| Category 5 (largest) | 1.31 (1.23-1.39) | | | <.0001 |
| Oral contraceptive use |  |  |  |  |
| Never | 1 (ref) | | | --- |
| Former | 1.00 (0.95-1.05) | | | .895 |
| Current | 1.55 (1.44-1.67) | | | <.0001 |
| Missing | 0.66 (0.55-0.81) | | | <.0001 |
| Menopausal status and HT use |  |  |  |  |
| Pre-menopausal | 1 (ref) | | | --- |
| Post-menopausal/never HT | 0.93 (0.87-0.99) | | | .022 |
| Post-menopausal/past HT | 0.93 (0.86-1.00) | | | .050 |
| Post-menopausal/current HT | 1.04 (0.99-1.10) | | | .129 |
| Post-menopausal/missing HT | 0.85 (0.68-1.05) | | | .125 |
| Unknown menopausal status | 1.14 (1.05-1.24) | | | .002 |
| Missing | 1.02 (0.94-1.10) | | | .676 |
| Acetaminophen use |  | | |  |
| No | 1 (ref) | | | --- |
| Yes | 1.24 (1.20-1.29) | | | <.0001 |
| Missing | 1.29 (1.10-1.51) | | | .002 |
| Aspirin use |  | | |  |
| No | 1 (ref) | | | --- |
| Yes | 1.18 (1.13-1.23) | | | <.0001 |
| Missing | 1.03 (0.90-1.19) | | | .637 |
| Other NSAID use |  | | |  |
| No | 1 (ref) | | | --- |
| Yes | 1.27 (1.23-1.31) | | | <.0001 |
| Missing | 1.62 (1.39-1.89) | | | <.0001 |
| Hypercholesterolemia |  | | |  |
| No | 1 (ref) | | | --- |
| Yes | 1.45 (1.40-1.50) | | | <.0001 |

*Note*. PTSD=posttraumatic stress disorder. HT=hormone therapy. NSAID=nonsteroidal anti-inflammatory drug.

Supplementary Table S3. Associations of potential mediator variables with trauma/PTSD symptom status and risk of incident hypertension

| Proposed Mediator | Association with Trauma/PTSD Symptom Status^a^ | | Association with Hypertension Risk^b^ | |
| --- | --- | --- | --- | --- |
|  | Wald Statistic (df) | *P* | Hazard Ratio  (95% Confidence Interval) | *P* |
| Body mass index, kg/m^2^ | χ^2^ (4)=254.26 | <.0001 |  |  |
| <18.5 |  |  | 1 (ref) | --- |
| 18.5-<21 |  |  | 0.92 (0.76-1.12) | .395 |
| 21-<23 |  |  | 1.30 (1.07-1.57) | .007 |
| 23-<25 |  |  | 1.77 (1.47-2.14) | <.0001 |
| 25-<27 |  |  | 2.36 (1.95-2.84) | <.0001 |
| 27-<30 |  |  | 3.44 (2.85-4.14) | <.0001 |
| 30+ |  |  | 5.64 (4.69-6.79) | <.0001 |
| Cigarette smoking | χ^2^ (4)=375.90 | <.0001 |  |  |
| Never |  |  | 1 (ref) | --- |
| Former smoker |  |  | 1.06 (1.02-1.10) | .001 |
| Current, 1-14 cigs/day |  |  | 1.02 (0.94-1.11) | .559 |
| Current, 15-24 cigs/day |  |  | 1.08 (0.98-1.18) | .123 |
| Current, 25+ cigs/day |  |  | 1.23 (1.07-1.41) | .003 |
| Missing |  |  | 1.12 (0.82-1.53) | .489 |
| Alcohol consumption, grams/day | χ^2^ (4)=39.22 | <.0001 |  |  |
| 0 |  |  | 1 (ref) | --- |
| 1-<5 |  |  | 0.86 (0.83-0.89) | <.0001 |
| 5-<10 |  |  | 0.73 (0.69-0.77) | <.0001 |
| 10-<20 |  |  | 0.86 (0.81-0.91) | <.0001 |
| 20+ |  |  | 1.14 (1.06-1.23) | .0004 |
| Missing |  |  | 1.08 (1.02-1.15) | .011 |
| Physical activity, MET hrs/wk | χ^2^ (4)=11.93 | .018 |  |  |
| <3 |  |  | 1 (ref) | --- |
| 3-<9 |  |  | 0.83 (0.79-0.87) | <.0001 |
| 9-<18 |  |  | 0.74 (0.70-0.77) | <.0001 |
| 18-<27 |  |  | 0.69 (0.65-0.73) | <.0001 |
| 27+ |  |  | 0.60 (0.57-0.63) | <.0001 |
| Alternative Healthy Eating Index | χ^2^ (4)=163.10 | <.0001 |  |  |
| 1^st^ quintile (worst diet) |  |  | 1 (ref) | --- |
| 2^nd^ quintile |  |  | 0.89 (0.84-0.93) | <.0001 |
| 3^rd^ quintile |  |  | 0.84 (0.81-0.88) | <.0001 |
| 4^th^ quintile |  |  | 0.74 (0.71-0.78) | <.0001 |
| 5^th^ quintile (best diet) |  |  | 0.62 (0.58-0.65) | <.0001 |
| Missing |  |  | 1.05 (0.84-1.32) | .669 |
| Sugar-sweetened beverage consumption | χ^2^ (4)=46.57 | <.0001 |  |  |
| <1/month |  |  | 1 (ref) | --- |
| 1-4/month |  |  | 1.03 (0.98-1.07) | .231 |
| 2-6/week |  |  | 1.03 (0.98-1.08) | .200 |
| 1+/day |  |  | 1.20 (1.14-1.26) | <.0001 |
| Missing |  |  | 1.19 (1.11-1.27) | <.0001 |
| Artificially-sweetened beverage consumption | χ^2^ (4)=25.41 | <.0001 |  |  |
| <1/month |  |  | 1 (ref) | --- |
| 1-4/month |  |  | 1.08 (1.02-1.14) | .009 |
| 2-6/week |  |  | 1.15 (1.10-1.20) | <.0001 |
| 1+/day |  |  | 1.38 (1.33-1.44) | <.0001 |
| Missing |  |  | 1.33 (1.24-1.43) | <.0001 |
| Antidepressant use | χ^2^ (4)=3470.33 | <.0001 |  |  |
| No |  |  | 1 (ref) | --- |
| Yes |  |  | 1.32 (1.27-1.38) | <.0001 |

*Note*. PTSD=posttraumatic stress disorder. MET hrs/wk=metabolic equivalent hours/week.

^a^Results of generalized estimating equations examining trauma/PTSD symptom status as a predictor of each potential mediator (trauma/PTSD symptom status and potential mediators were time-varying variables that were updated over the course of the study period). The Wald statistic indicates whether there were significant differences in a given mediator among the different trauma/PTSD symptom status groups. Compared to no trauma exposure, elevated PTSD symptoms were associated with greater likelihood of having higher body mass index, smoking cigarettes, and using antidepressants. Elevated PTSD symptoms were also associated with lower alcohol, sugar-sweetened beverage, and artificially-sweetened beverage consumption, lower physical activity, and lower likelihood of having poor diet quality compared to no trauma exposure.

^b^Results of Cox proportional hazards models examining each potential mediator as a predictor of incident hypertension (adjusted for demographics, family history, and childhood adiposity).
