# Supplementary material for: A 10-year cohort analysis of routine paediatric ART data in a rural South African setting
Source: Epidemiol Infect. 2016 Sep 9;145(1):170–80. doi: 10.1017/S0950268816001916 (PMC5197927; doi:10.1017/S0950268816001916)
Supplement: Supplementary file 1 [file S0950268816001916sup.zip › S0950268816001916sup001.zip!/Supplementary_Fig S1_(Lilian).docx]

***(a)***

*p <0.0001*

*p <0.0001*

Over 5 yrs of age at ART initiation

***(b)***

*p <0.0001*

Excluding early losses to follow-up at 0.001 yrs

***(c)***

*p <0.0001*

Supplementary Fig. S1. Retention in care to 5 years on treatment by a) treatment initiation period, stratified by age at ART initiation, b) baseline regimen excluding early losses to follow-up at 0.001 years and c) viral suppression in children with a recent viral load test receiving 6 months or more of ART (p = Log-rank test).
A3E, Abacavir Lamivudine Efavirenz; A3L, Abacavir Lamivudine Lopinavir; ART, Antiretroviral Therapy; LTFU, Losses to Follow-Up; S3E, Stavudine Lamivudine Efavirenz; S3L, Stavudine Lamivudine Lopinavir
