# Supplementary material for: A 10-year cohort analysis of routine paediatric ART data in a rural South African setting
Source: Epidemiol Infect. 2016 Sep 9;145(1):170–80. doi: 10.1017/S0950268816001916 (PMC5197927; doi:10.1017/S0950268816001916)
Supplement: Supplementary file 1 [file S0950268816001916sup.zip › S0950268816001916sup001.zip!/Supplementary_Table S1_(Lilian).docx]

Supplementary Table S1. *Children who transferred out of the ART programme, excluded from the cohort analysis, compared to the analysed cohort*

|  | Analysed Cohort | Transferred Out | p |
| --- | --- | --- | --- |
|  |  |  |  |
| n | 5461 | 1345 |  |
| Gender, n (%) |  |  | 0.126 |
| Male | 2689 (49.2%) | 631 (46.9%) |  |
| Female | 2772 (50.8%) | 714 (53.1%) |  |
| ART initiation period, n (%) |  |  | **<0.001**† |
| 1 (<Apr 2010) | 1720 (31.5%) | 647 (48.1%) |  |
| 2 (Apr 2010-Aug 2012) | 1897 (34.7%) | 551 (41.0%) |  |
| 3 (>Aug 2012) | 1844 (33.8%) | 147 (10.9%) |  |
| Age at initiation (years), median (range) | 5.0 (0.001-15.0) | 5.1 (0.001-15.0) | 0.463 |
| On TB treatment at ART initiation,  n (%) |  |  | 0.109 |
| Yes | 348 (7.5%) | 107 (8.8%) |  |
| No | 4320 (92.5%) | 1104 (91.2%) |  |
| Baseline CD4 count (cells/mm^3^), median (range) | 259 (0-3000) | 154 (1-2747) | **<0.0001** |
| Baseline regimen in children ≤3 years of age at ART initiation, n (%) |  |  | **<0.001** |
| A3L | 1072 (80.3%) | 166 (67.5%) |  |
| S3L | 263 (19.7%) | 80 (32.5%) |  |
| Baseline regimen in children >3 years of age at ART initiation, n (%) |  |  | **<0.001** |
| A3E | 1533 (52.2%) | 239 (32.2%) |  |
| S3E | 1402 (47.8%) | 503 (67.8%) |  |
|  |  |  |  |

A3E, Abacavir Lamivudine Efavirenz; A3L, Abacavir Lamivudine Lopinavir; ART, Antiretroviral Therapy; S3E, Stavudine Lamivudine Efavirenz; S3L, Stavudine Lamivudine Lopinavir; TB, Tuberculosis. Statistically significant differences are shown in bold.
† Significant difference between ART initiation periods 1 and 2 (p <0.001), 1 and 3 (p <0.001) and 2 and 3 (p <0.001)
